# Supplementary material for: TTYH family members form tetrameric complexes at the cell membrane
Source: Commun Biol. 2022 Aug 30;5:886. doi: 10.1038/s42003-022-03862-3 (PMC9427776; doi:10.1038/s42003-022-03862-3)
Supplement: Supplementary file 2 — Supplementary Information [file 42003_2022_3862_MOESM2_ESM.pdf]

Supplementary information

TTYH family members form tetrameric complexes at the cell membrane

Emelia Melvin[1], Zuzana Kalaninová[2,3], Elia Shlush[1], Petr Man[2], Moshe Giladi[1,4,*] and Yoni Haitin[1,5,*]

Supplementary Fig. 1. *In situ* cross-linking analysis of 14-3-3γ.

Supplementary Fig. 2. Subunit counting data binomial fit.

Supplementary Fig. 3. Thermal denaturation of mTTYH paralogs.

Supplementary Fig. 4. HDX profiles of the mTTYH1 at 25 ºC.

Supplementary Fig. 5. HDX profiles of the GFP fusion.

Supplementary Fig. 6. The effect of 2-mercaptoethanol (2-ME) on LMNG-solubilized mTTYH1 and mTTYH3.

Supplementary Movie 1. TIRF microscopy movie of an oocyte expressing EGFP-fused mTTYH3.

Supplementary Table 1. List of primers used in this study.

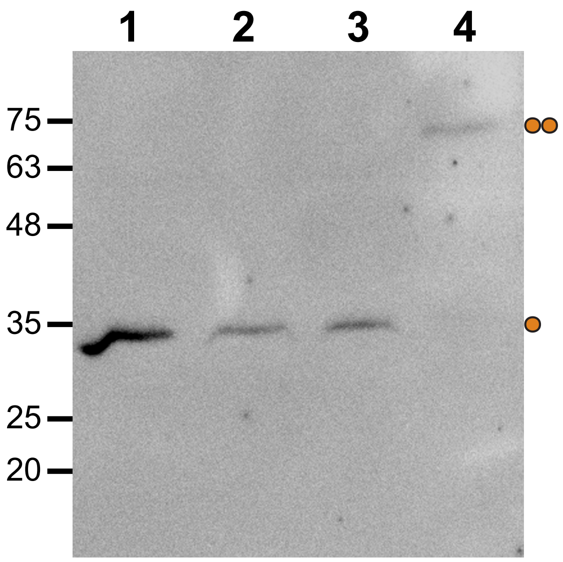

Supplementary Fig. 1. *In situ* cross-linking analysis of 14-3-3γ. Western blot analysis of HEK 293 cell lysates expressing C-terminally myc-tagged 14-3-3γ. Following treatment with PBS (1), 2000 μM of the membrane-impermeable cross-linker BS^3 (2), DMSO (3), and 2000 μM of the membrane-permeable DSS (4), cells were lysed and subjected to immunoblot detection using an anti-myc antibody.

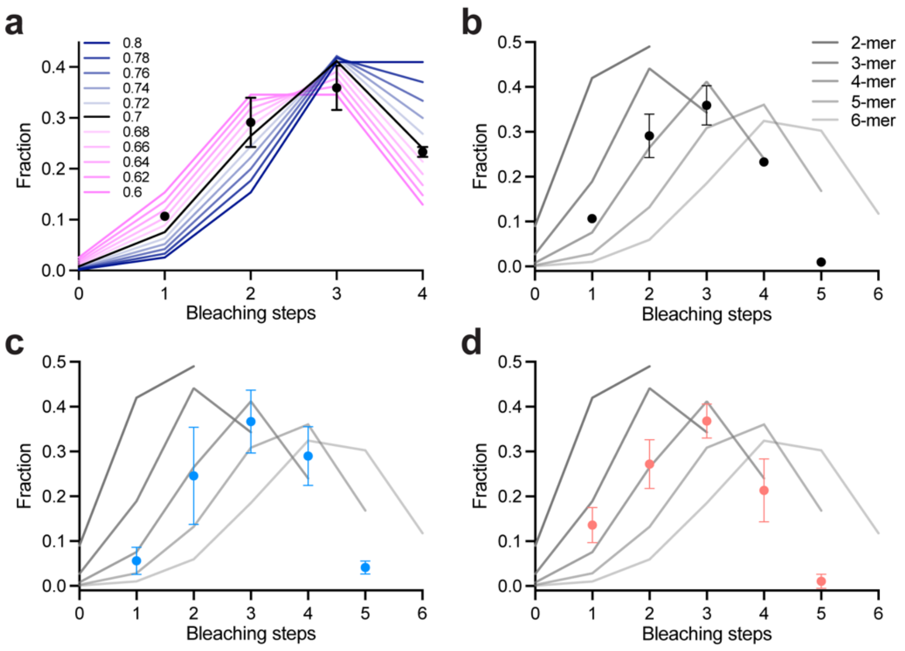

Supplementary Fig. 2. Subunit counting data binomial fit. a Determination of the EGFP probability of being fluorescent (*p*). Data collected from oocytes expressing homotetrameric KCNH1 channels was fitted with the expected distribution of tetrameric channels containing 1, 2, 3 or 4 functional GFP tags, allowing the probability that the EGFP tag is fluorescent to be a free parameter. A probability of 0.7 of the EGFP to be fluorescent matches well the observed data. **b-d** Distribution of the number of bleaching steps observed from oocytes expressing KCNH1 (**b**), mTTYH1 (**c**), and mTTYH3 (**d**). Circles, the average percentage of spots that bleached in each number of bleaching steps. Binomial equation fits, assuming the number of monomers in the complex and with the percentage of fluorescent EGFP molecules being a free parameter (graded grey shaded lines).

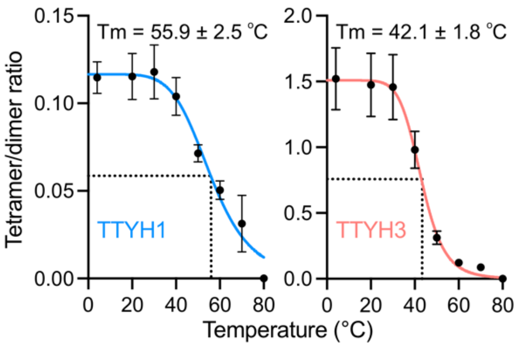

Supplementary Fig. 3. Thermal denaturation of mTTYH paralogs. Melting curves of mTTYH1 (left) and mTTYH3 (right). Melting temperatures (Tm) for tetramer/dimer ratio were calculated by fitting the curves with the Boltzmann sigmoidal equation (n = 3).

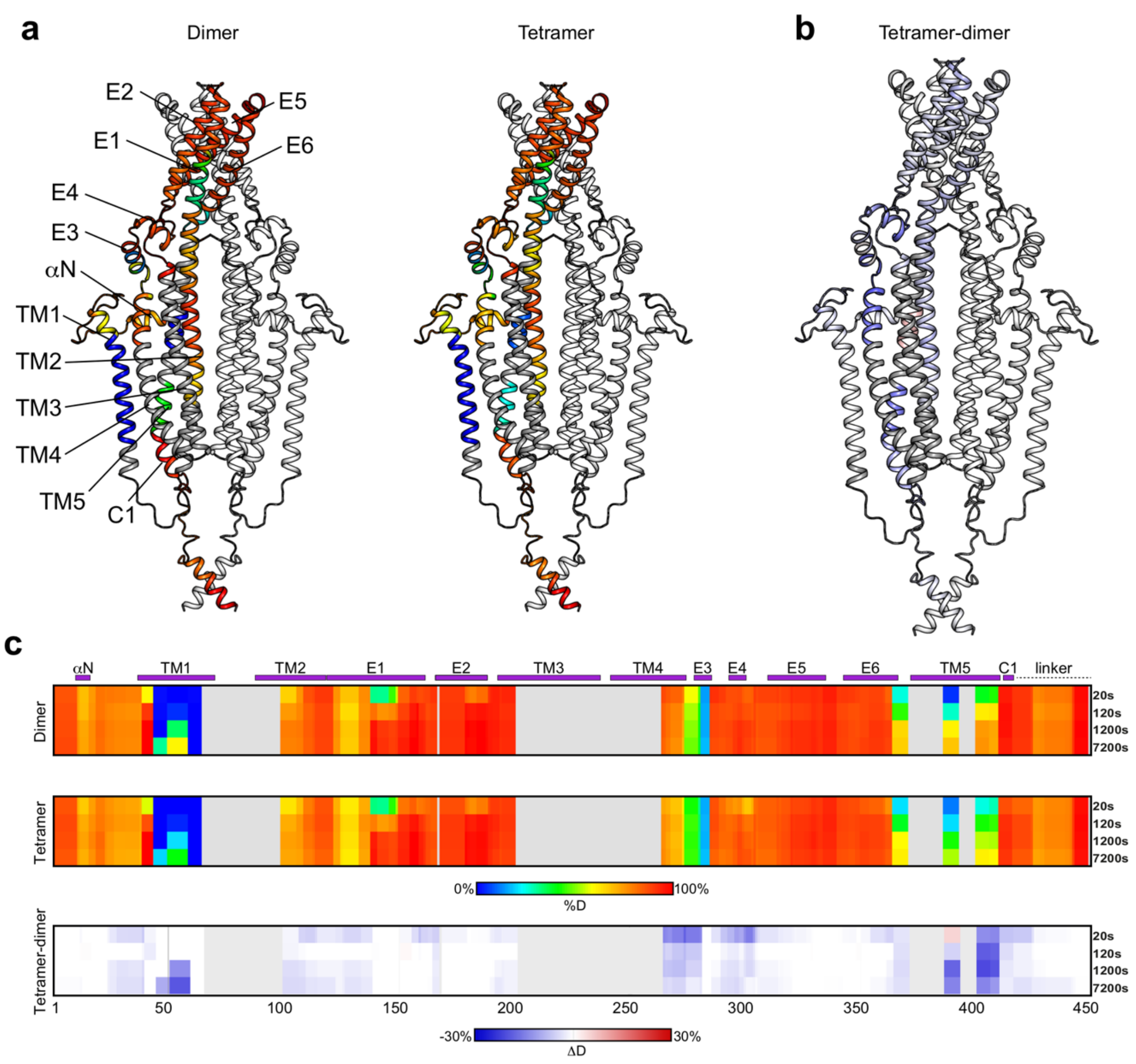

Supplementary Fig. 4. HDX profiles of the mTTYH1 at 25 ºC. a Cartoon representations of mTTYH1 homology model. A single chain is colored as a heat map, representing the HDX level following 20 sec incubation in D_2O for the dimeric (left) and tetrameric (right) populations at 25 ºC. The highest and lowest exchange levels indicated in red and blue, respectively. **b** The HDX difference between the tetrameric and dimeric populations, following 20 sec incubation in D_2O is mapped onto the mTTYH1 homology model. **c** Deuteration levels at the indicated time points for the dimer (upper panel) and tetramer (middle panel). The difference between the tetrameric and dimeric populations is shown (bottom panel). The secondary structures elements are indicated above the heat maps.

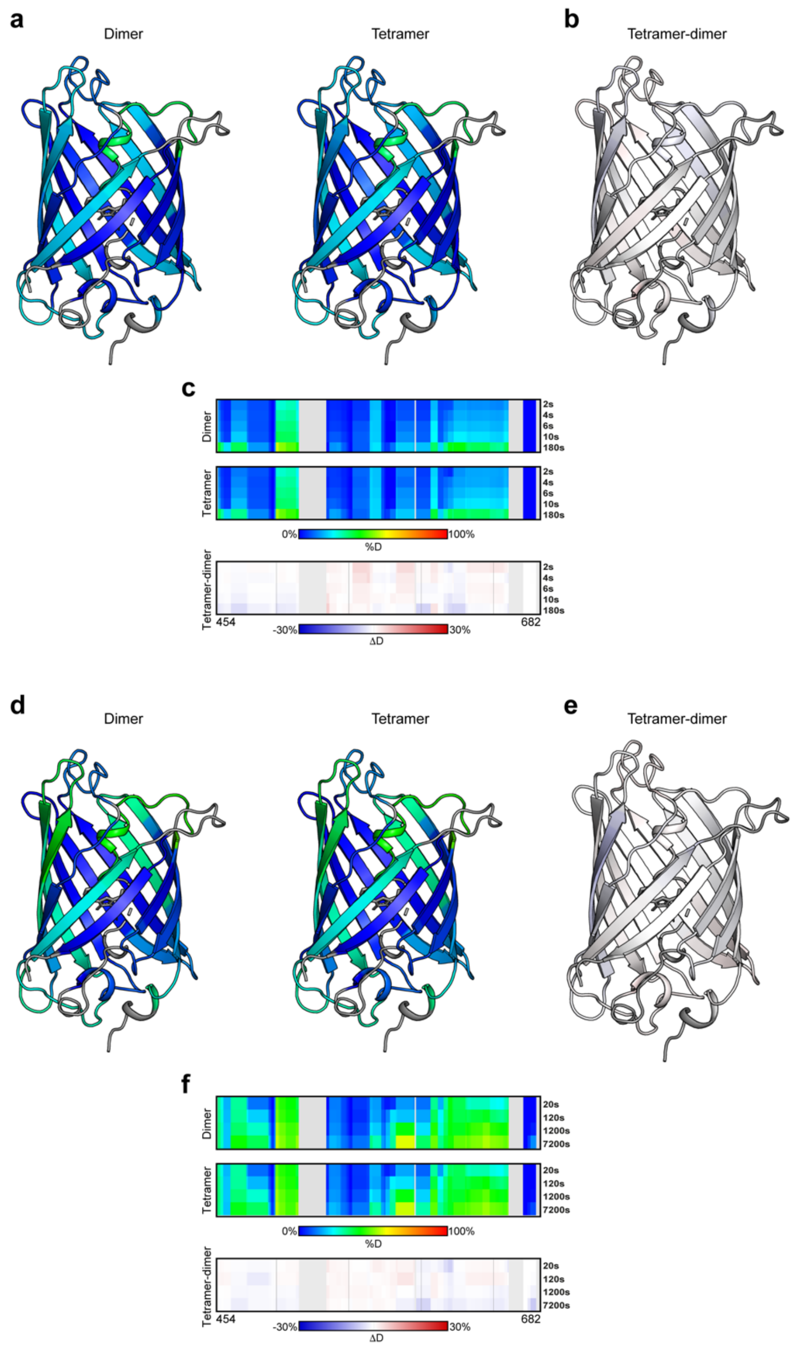

Supplementary Fig. 5. HDX profiles of the GFP fusion. a Cartoon representations of GFP, colored as a heat map representing the HDX level following 10 sec incubation in D_2O for the dimeric (left) and tetrameric (right) mTTYH1 populations. The highest and lowest exchange levels indicated in red and blue, respectively. **b** The HDX difference between the GFP fusions of the tetrameric and dimeric populations, following 10 sec incubation in D_2O is mapped onto the GFP structure. **c** Deuteration levels at the indicated time points for the dimer (upper panel) and tetramer (middle panel) GFP fusions. The difference between the tetrameric and dimeric populations GFP fusions is shown (bottom panel).

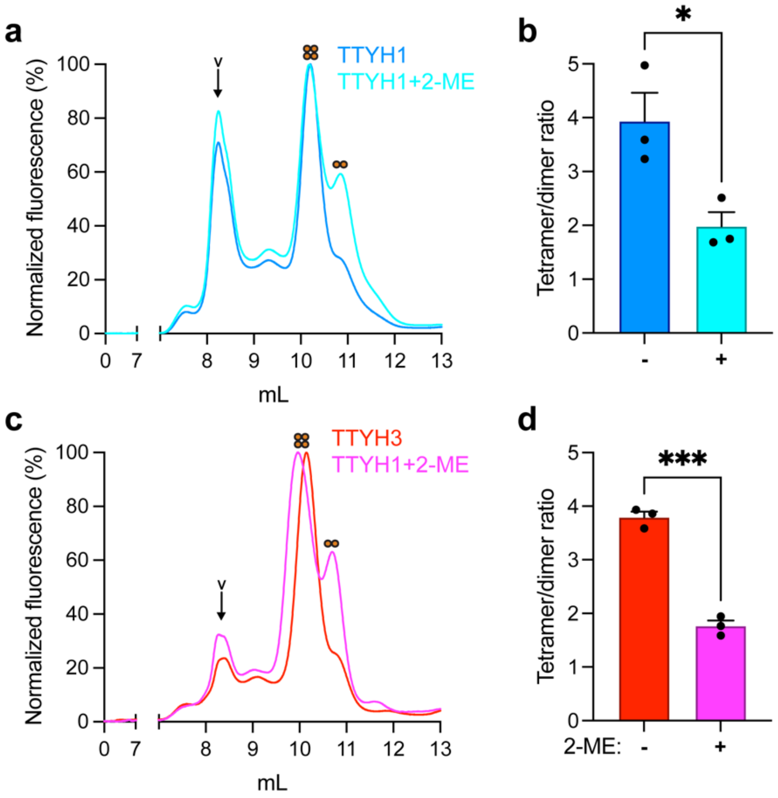

Supplementary Fig. 6. The effect of 2-mercaptoethanol (2-ME) on LMNG-solubilized mTTYH1 and mTTYH3. a,c Representative FSEC elution profiles of mTTYH1 (**a**) and mTTYH3 (**c**) in the absence or presence of 2-ME in the mobile phase, as indicated. **b,d** Tetramer/dimer ratio of the mTTYH1 (**b**) and mTTYH (**d**) in accordance with the 2-ME treatment, as indicated. (n=3, * $p < 0.05$; *** $p < 0.01$).

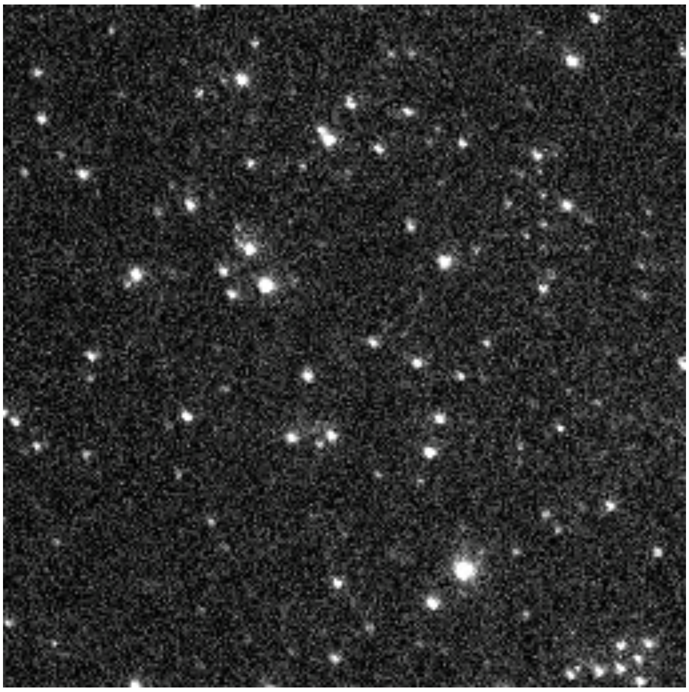

Supplementary Movie 1. TIRF microscopy movie of an oocyte expressing EGFP-fused mTTYH3. The full movie recording corresponding to the representative frame presented in Fig. 2d is provided.

Primer name	Primer sequence	Used to
pEGFP-N1 F	GCCACCATGGTGAGCAAG	Generate of mTTYH1/3.pEGFP-N1
pEGFP-N1 R	GGATCTGACGGTTCACTAAAC	Generate of mTTYH1/3.pEGFP-N1
mTTYH3 FLAG F	GATGATGATAAATAAAGCGGCCGCGACTCTAGATCATAATCAGC	Replace EGFP with FLAG tag (mTTYH3)
mTTYH3 FLAG R	ATCTTTATAATCGGTGGCGTGCCCGCTGCC	Replace EGFP with FLAG tag (mTTYH3)
mTTYH3 F	TTTAGTGAACCGTCAGATCCATGGCCGGGGTCAGCTACG	Insert mTTYH3 into pEGFP-N1
mTTYH3 R	CCCTTGCTCACCATGGTGGCGTGCCCGCTGCCGCTGGA	Insert mTTYH3 into pEGFP-N1
mTTYH1 FLAG F	GATGATGATAAATAAAGCGGCCGCGACTCTAG	Replace EGFP with FLAG tag (mTTYH1)
mTTYH1 FLAG R	ATCTTTATAATCGGTGGCGACCGGTCCGAT	Replace EGFP with FLAG tag (mTTYH1)
8HIS mTTYHEGFP F	CACCACCACCACTAAAGCGGCCGCGACTCTA	Insert 8xHIS downstream of EGFP in mTTYH.pEGFP-N1
8HIS mTTYHEGFP R	ATGATGATGATGCTTGTACAGCTCGTCCATGC	Insert 8xHIS downstream of EGFP in mTTYH.pEGFP-N1
GH19 R	CCTCCCGACTGCCGGACT	Linearize GH19
GH19 F	TGAGAGGATCATTTCAAAAC	Linearize GH19
mTTYH3 EGFP GH19 F	AGAGTCCGGCAGTCGGGAGGATGGCCGGGGTCAGCTAC	Insert mTTYH3 EGFP into GF19
mTTYH3 EGFP GH19 R	GTTTTGAAATGATCCTCTCACTTGTACAGCTCGTCCATGC	Insert mTTYH1/3 EGFP into GF19
mTTYH1 EGFP GF19 F	AGAGTCCGGCAGTCGGGAGGATGGGGGCACCCCCGGGC	Insert mTTYH1 EGFP into GF19
GFPHis pFastBac F	TCTAAAGGTGAAGAATTATTCACTGGCGTTGTCC	Linarize GFPHis pFastBac without the ATG
GFPHis pFastBac R	GGGGGGGGATCCGCGCCCGA	Linarize GFPHis pFastBac without the ATG
mTTYH1 into GFPHis pFastBac F	TCGGGCGCGGATCCCCCCCCATGGGGGCACCCCCGGGC	Insert mTTYH1 into GFPHis pFastBac
mTTYH1 into GFPHis pFastBac R	AATAATTCTTCACCTTTAGAGATGGAAGACTGCCACTGCACAAAGCG TTTGG	Insert mTTYH1 into GFPHis pFastBac
mTTYH3 ECDs Hifi vec F	GGCAGCGAGCTGGCTGTGTCT	Generate mTTYH3 ECD1-ECD2
mTTYH3 ECDs Hifi vec R	TCCCATGGGTATATCTCCTTCTTAAAG	Generate mTTYH3 ECD1-ECD2
mTTYH3 ECDs Hifi ins F	GAAGGAGATATACCCATGGGAAACGGCGAGACCAGCGATGGC	Generate mTTYH3 ECD1-ECD2
mTTYH3 ECDs Hifi ins R	AGACACAGCCAGCTCGCTGCCGCTGCCCCTGTACCAGTCATAGAG	Generate mTTYH3 ECD1-ECD2
mTTYH3 ECDs for T4L R	CCTGTACCAGTCATAGAGGTCC	Linearize mTTYH3 ECD1-ECD2
mTTYH3 ECDs for T4L F	GAGCTGGCTGTGTCTGTG	Primer to linearize mTTYH3 ECD1-ECD2
T4L for mTTYH3 R	AGACACAGCCAGCTCGGCGGCATACGCGTCCCA	Insert T4L between ECDs
T4L for mTTYH3 F	CTATGACTGGTACAGGGCCGCCAATATATTTGAAATGTTACGTATAG AT	Insert T4L between ECDs
mTTYH3 C271A F	CAGCGACTTCGCCGTAGACCCTGACAC	Generate C271A
mTTYH3 C271A R	GAGCCCACAGACACAGCC	Generate C271A
mTTYH3 C299A F	CTACTTGGCTGCCTCACCCCGTG	Generate C299A
mTTYH3 C299A R	TATTGCAAAATGTCCCCAC	Generate C299A
mTTYH3 C366A F	CCTGGTGGACGCCCGCAGCTTGC	Generate C366A
mTTYH3 C366A R	GCGGTGAGGTGCTGGAGA	Generate C366A
mTTYH3 CC381A F	GACAGGCTTCGCCTATGATGGTGTGGAG	Generate C381A
mTTYH3 CC381A R	AGAGCCTGCACGTAGTCC	Generate C381A

Supplementary Table 1. List of primers used in this study.
