# Supplementary material for: TTYH family members form tetrameric complexes at the cell membrane
Source: Commun Biol. 2022 Aug 30;5:886. doi: 10.1038/s42003-022-03862-3 (PMC9427776; doi:10.1038/s42003-022-03862-3)
Supplement: Supplementary file 3 — Description of Additional Supplementary Data [file 42003_2022_3862_MOESM3_ESM.pdf]

Description of Additional Supplementary Files

File name: Supplementary Data 1

Description: Source data for Figs. 2c and 5c, and Supplementary Fig. 2,3, and 6 are available in Supplementary Data 1.

File name: Supplementary Data 2

Description: Uncropped gel images corresponding to Figs. 1a-c, 3a, 6b, 6c, and Supplementary Fig. 1.

File name: Supplementary Movie 1

Description: TIRF microscopy movie of an oocyte expressing EGFP-fused mTTYH3.
