# Supplementary material for: TTYH family members form tetrameric complexes at the cell membrane
Source: Commun Biol. 2022 Aug 30;5:886. doi: 10.1038/s42003-022-03862-3 (PMC9427776; doi:10.1038/s42003-022-03862-3)
Supplement: Supplementary file 7 — Reporting Summary [file 42003_2022_3862_MOESM7_ESM.pdf]

nature portfolio

Corresponding author(s): Yoni Haitin and Moshe Giladi

Last updated by author(s): Jul 25, 2022

Reporting Summary

Nature Portfolio wishes to improve the reproducibility of the work that we publish. This form provides structure for consistency and transparency in reporting. For further information on Nature Portfolio policies, see our Editorial Policies and the Editorial Policy Checklist.

Statistics

For all statistical analyses, confirm that the following items are present in the figure legend, table legend, main text, or Methods section.

n/a	Confirmed	
☐	☒	The exact sample size (*n*) for each experimental group/condition, given as a discrete number and unit of measurement
☐	☒	A statement on whether measurements were taken from distinct samples or whether the same sample was measured repeatedly
☐	☒	The statistical test(s) used AND whether they are one- or two-sided<br>*Only common tests should be described solely by name; describe more complex techniques in the Methods section.*
☒	☐	A description of all covariates tested
☒	☐	A description of any assumptions or corrections, such as tests of normality and adjustment for multiple comparisons
☐	☒	A full description of the statistical parameters including central tendency (e.g. means) or other basic estimates (e.g. regression coefficient) AND variation (e.g. standard deviation) or associated estimates of uncertainty (e.g. confidence intervals)
☐	☒	For null hypothesis testing, the test statistic (e.g. *F*, *t*, *r*) with confidence intervals, effect sizes, degrees of freedom and *P* value noted<br>*Give P values as exact values whenever suitable.*
☒	☐	For Bayesian analysis, information on the choice of priors and Markov chain Monte Carlo settings
☒	☐	For hierarchical and complex designs, identification of the appropriate level for tests and full reporting of outcomes
☒	☐	Estimates of effect sizes (e.g. Cohen's *d*, Pearson's *r*), indicating how they were calculated

Our web collection on statistics for biologists contains articles on many of the points above.

Software and code

Policy information about availability of computer code

Data collection	Shimadzu LabSolutions 5.82
Data analysis	Shimadzu LabSolutions 5.82, Prism 9 9.3.1 (350), UCSF chimera 1.14, PyMol 2.0, Bruker Daltonics data analysis, MASCOT search engine 2.7, DeuteEx, MSTools

For manuscripts utilizing custom algorithms or software that are central to the research but not yet described in published literature, software must be made available to editors and reviewers. We strongly encourage code deposition in a community repository (e.g. GitHub). See the Nature Portfolio guidelines for submitting code & software for further information.

Data

Policy information about availability of data

All manuscripts must include a data availability statement. This statement should provide the following information, where applicable:

- Accession codes, unique identifiers, or web links for publicly available datasets
- A description of any restrictions on data availability
- For clinical datasets or third party data, please ensure that the statement adheres to our policy

The mass spectrometry proteomics data have been deposited to the ProteomeXchange Consortium via the PRIDE partner repository with the dataset identifier PXD031833. Source data are provided with this paper. Any other relevant data are available from corresponding authors upon request.

Field-specific reporting

Please select the one below that is the best fit for your research. If you are not sure, read the appropriate sections before making your selection.

☒ Life sciences ☐ Behavioural & social sciences ☐ Ecological, evolutionary & environmental sciences

For a reference copy of the document with all sections, see nature.com/documents/nr-reporting-summary-flat.pdf

Life sciences study design

All studies must disclose on these points even when the disclosure is negative.

Sample size	No statistical methods were used to predetermine sample sizes. Required experimental sample sizes were chosen according to common practice in protein biochemistry and microscopy (at least three independent experiments). Statistical analysis was limited to determine mean ± SEM.
Data exclusions	Data were not excluded.
Replication	Subunit counting analyses were recorded from multiple oocytes. Recordings were replicated on multiple occasions from different expressing cells, and all replications were included in the analyses. FSEC, cross-linking and blue-native PAGE analyses were performed in triplicates consisting of different transfections, performed on different occasions. For the HDX-MS experiments, the 25 °C experiment was performed at four time points (20 seconds, 2 minutes, 20 minutes and 2 hours), with two points (20 seconds and 20 minutes) replicated (n=3). The 4 °C experiments was performed at four time points (2,4,6, 10, and 180 seconds), with the 2 and 6 seconds time points replicated (n=3). All replications were successful and included in the presented data.
Randomization	Randomization was not performed in this study as samples were predefined and did not require allocation into different experimental groups. The groups were prepared independently and compared under controlled conditions.
Blinding	Blinding was not performed in this study as the results are quantitative in nature and do not require subjective interpretation.

Reporting for specific materials, systems and methods

We require information from authors about some types of materials, experimental systems and methods used in many studies. Here, indicate whether each material, system or method listed is relevant to your study. If you are not sure if a list item applies to your research, read the appropriate section before selecting a response.

Materials & experimental systems

n/a	Involved in the study	
☐	☒	Antibodies
☐	☒	Eukaryotic cell lines
☒	☐	Palaeontology and archaeology
☒	☐	Animals and other organisms
☒	☐	Human research participants
☒	☐	Clinical data
☒	☐	Dual use research of concern

Methods

n/a	Involved in the study	
☒	☐	ChIP-seq
☒	☐	Flow cytometry
☒	☐	MRI-based neuroimaging

Antibodies

Antibodies used	The antibodies used in this study are commercially available and include the anti-FLAG M2 monoclonal IgG (Sigma-Aldrich; F3165), HRP-conjugated anti-mouse polyclocnal IgG (Jackson ImmunoResearch Inc.; 115-035-003)
Validation	All antibodies used were validated by the suppliers and the validation report is available from the suppliers websites (https://www.sigmaaldrich.com/IL/en/product/sigma/f3165; https://www.jacksonimmuno.com/catalog/products/115-035-003)

Eukaryotic cell lines

Policy information about cell lines

Cell line source(s)	HEK293T (ATCC; CLR-1573), Sf9 (ThermoFisher Scientific; 11496015).
Authentication	No further authentication was performed for commercially available cell lines. Authetication available online: https://www.atcc.org/products/crl-1573; https://www.thermofisher.com/order/catalog/product/11496015.
Mycoplasma contamination	Cell lines were not tested for mycoplasma contamination.

Commonly misidentified lines (See ICLAC register)	No commonly misidentified cell lines were used in this study.
